# Anti Müllerian Hormone (AMH) level and expression in mural and cumulus cells in relation to age

**DOI:** 10.1186/s13048-014-0113-3

**Published:** 2014-12-11

**Authors:** Alon Kedem, Yuval Yung, Gil M Yerushalmi, Jigal Haas, Ettie Maman, Mirit Hanochi, Rina Hemi, Raoul Orvieto, Jehoshua Dor, Ariel Hourvitz

**Affiliations:** IVF unit, Department of Obstetrics and Gynecology, Sheba Medical Center, Tel Hashomer, affiliated with the Sackler Faculty of Medicine, Tel Aviv University, Tel Aviv, Israel; Institute of Endocrinology, Sheba Medical Center, Tel Hashomer, affiliated with the Sackler Faculty of Medicine, Tel-Aviv University, Tel-Aviv, Israel

## Abstract

**Background:**

Serum AMH is declining with age and is highly associated with ovarian follicular reserve and disordered folliculogenesis. However, the precise role of AMH in the process of human follicular aging has still to be determined.

**Aim:**

This study investigates AMH level in the follicular fluid (FF) and mRNA expression pattern in cumulus and mural granulosa cells of human ovarian follicles in relation to age.

**Methods:**

We conducted a prospective study. Sixty-eight women undergoing In vitro fertilization (IVF) treatment were enrolled in the study. We obtained FF, mural and cumulus granulosa cells from large preovulatory follicles (17-20 mm) of 21–35 years old women (n = 40) and 40–45 years old women (n = 28) during oocyte pickup.

**Results:**

Higher level of AMH mRNA expression in cumulus cells was observed in the older age group compared to the younger (P <0.01). In accordance with AMH mRNA expression results, FF AMH protein levels were significantly higher in the older group than in the younger group (4.7 ± 1.1 ng\ml and 2.3 ± 0.2 ng\ml respectively, p < 0.002).

**Conclusions:**

AMH is highly expressed and secreted from cumulus GCs of advanced age patients. This remarkable correlation between AMH mRNA levels in cumulus cells in respect to age suggests that AMH may be involved in follicular aging process.

## Introduction

The final phase of ovarian follicular development is a complex process occurring within a characteristic hormonal microenvironment of the follicular fluid. The completion of oocyte maturation coinciding with the development and growth of the antral follicles is under the control of endocrine, paracrine and autocrine control.

Anti-Müllerian hormone (AMH) is a dimeric glycoprotein and a member of the transforming growth factor-beta superfamily [1]. The role of AMH as an important regulator of mammalian follicular development has recently been described [2,3]. In females AMH is produced exclusively by granulosa cells (GCs) of ovarian follicles [4]. It is normally expressed at low levels in primary follicles, increases to maximal levels in large preantral and small antral follicles and then declines as the follicle grows [5-9].

Ovarian aging is characterized by gradual decrease in oocyte quality and quantity. Although the rate of follicle disappearance has been extensively investigated as reported by Faddy et al. [10], age-related cellular and molecular aspects of the follicle pool are still poorly defined. It is widely accepted that apoptosis is the driving force behind follicle loss with ageing [11] a condition suggesting the occurrence of specific age-related alterations in the oocyte and granulosa cells.

Recently, we have demonstrated a pathological dysregulation of AMH expression and secretion in antral follicles from atretic and immature oocytes [12] and in follicles of PCOS patients [5]. These data emphasize the association between the physiological down regulation of AMH and follicular antral health. Since the possible relationship between intrafollicular AMH within an individual preovulatory follicle and patient age has not been determined, in the present study we aim to investigate AMH level in the follicular fluid (FF) and mRNA expression pattern in cumulus and mural GCs of human ovarian follicles in relation to age.

## Methods

### Patients

The Ethics Committee of Sheba Medical Centre, Tel Hashomer approved the study protocol, and written consent (approved by the IRB) was obtained from all participants (Reference number 920110559). From March 2009 to October 2013, Sixty-eight women undergoing IVF treatment by standard ovarian stimulation protocols were prospectively studied. The patients’ characteristics are described in Table 1. All women met the following criteria: (i) Age: 21–35 years old or 40–45 years old (ii) normovulatory; (iii) no clinical signs of hyperandrogenism; and (iiii) body mass index 20–31 kg/m2. Patients with polycystic ovary syndrome were excluded.

**Table 1:** **Characteristics of 68 patients participating in the study**

	**21-35 years**	**40-45 years**	**P**
Patients No.	40	28	
Age (years)	31 ± 2.1	42 ± 1	
BMI	23 ± 2.8	24 ± 3.1	NS
AMH	2.3 ± 2.8	0.7 ± 0.6	0.01
FSH (IU)	6.9 ± 1.7	9.5 ± 5	0.02
17-β Estradiol (picomol/L)	1395 ± 1155	1237 ± 928	NS
**Total gonadotropin dosage (I.U) (mean ± SD)**	1800 ± 1875	2850 ± 1950	0.02
Mean no. Of collected oocytes	9 ± 6	5 ± 3	0.01

### IVF protocol

All women were down regulated with gonadotropin-releasing hormone agonist (Decapeptyl 0.1 mg, Ferring Co, Kiel, Germany), starting on cycle day 21. After 10–14 days, ovarian stimulation was carried out with recombinant FSH (Gonal F Serono, Aubonne, Switzerland) or human menopausal gonadotropin (hMG) (Menogon, Ferring, Germany).

The initial dose of ovarian stimulation was dependent upon patient age, body mass index and prior treatment history. When three leading follicles reached 18 mm in diameter, patients received human chorionic gonadotropin (Ovitrelle 250 mg; Merck Serono). Oocyte retrieval was performed with transvaginal ultrasound-guided needle aspiration.

### AMH measurements in serum and FF

FF from one leading preovulatory follicles (diameter 17–20 mm) per patient was individually aspirated using a 10-ml syringe under transvaginal ultrasound guidance. Follicular fluid (without any diluting fluid) from each individual follicle was separated from the oocyte and the needle was thoroughly washed before the aspiration of the next follicle. After ensuring that no oocytes were left, all flushing volumes were discarded. This methodology was used to ensure complete separation between FF and to minimize blood contamination. After oocyte isolation, FF were centrifuged at 500 g for 15 min at 4_C and the supernatants were collected and stored at _80_C for future hormonal analysis. Serum AMH levels were measured prior to all subsequent treatment cycles. FF and serum AMH concentrations were measured by enzyme-linked immunosorbent assay (DSL-10–14400; Diagnostic Systems Laboratory, Webster, TX, USA). The intra- and inter-assay coefficients of variations were 4.6 and 8.0%, respectively.

### Cumulus and Mural GC extraction and purification

Granulosa cells and FF for protein level were obtained from the same follicles. Cumulus GCs were obtained during oocyte denudation for ICSI. Briefly, after oocyte retrieval, the cumulus cells were removed from the cumulus–oocyte–complex with hyaluronidase. The cells were centrifuged at 200 g for 5 min at room temperature and the resulting pellets were subjected to RNA extraction.

Mural GCs were collected from FFs under a microscope, carefully avoiding blood clots, and re-suspended in IVF medium or phosphate buffered solution (PBS) as described elsewhere [13].

### RNA extraction, reverse transcription and RT-PCR

Total RNA was extracted from GCs by the Mini RNA Isolation I Kit (Zymo Research Corp., CA). Total RNA (100 ng) from each sample was used for cDNA synthesis using High capacity RT-PCR Kit (Applied Biosystems, Carlsbad, CA) according to manufacturer’s instructions. Real-time PCR reaction mix contained cDNA was done as described earlier [12]. See Table 2 for primer sequences.

**Table 2:** **Real-time PCR primer sequences**

**Gene**	**Primer sequences**	**Product length**	**Acce No.**
AMH	Sense 5’- GCTGCCTTGCCCTCTCTAC	117	NM_000479.3
	Antisense, 5’- GAACCTCAGCGAGGGTGTT		
β-actin	Sense, 5’- CCTGGACTTCGAGCAAGAGA	117	NM_001101.3
	Antisense, 5’- CAGCGGAACCGCTCATTGCCAATGG		

### Statistics

Paired comparisons were performed using the unpaired two-sided Student’s *t*-test assuming unequal variances. All statistical procedures were run on PASW Statistics version 18.0.0 (SPSS).

## Results

### Patient characteristics

Forty women 21–35 years old and 28 women 40–45 years old met our inclusion criteria. Patient characteristics are described in Table 1.

### AMH mRNA expression from cumulus and mural granulosa cells in relation to age

To study AMH expression and secretion during late antral ovarian follicle development in relation to age, we obtained cumulus GCs, mural GCs and FF from the leading follicle of each patient. The results are an average of 4 assays of rt PCR. In each assay 5–8 patient were included in each assay.

**Figure 1:**
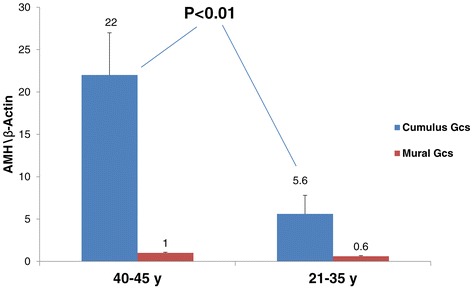
**AMH mRNA expression in cumulus and mural granulosa cells in relation to age.** Total mRNA was extracted from cumulus and granulosa GCs related to age. The RNAs were subjected to reverse transcription and qRT-PCR for AMH and β-actin. The results are expressed as mean + SEM of four assays, 5 to 8 patients in each group.

Interestingly, we observed higher AMH mRNA expression in cumulus cells from the older age group (40–45 y) than from the younger group (21–35 y) (22.3 ± 5.1vs 5.6 ± 2.2 AMH\β-Actin, P <0.01 respectively) (Figure 1). In addition, AMH mRNA expression in cumulus GCs was significantly higher than in mural granulosa cells in both age groups (Figure 1). Furthermore, AMH mRNA expression in mural GCs was not significantly different between both age groups (1 ± 0.08 AMH\β-Actin in the older group and 0.6 ± 0.1 AMH\β-Actin in the younger group).

### FF AMH protein level in relation to age

**Figure 2:**
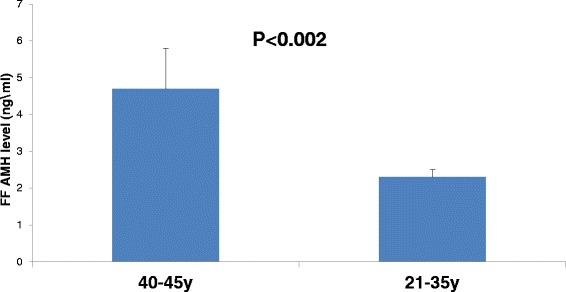
**FF AMH protein levels in relation to age.** We obtained FF from single dominant follicle of patients 40–45 years (n = 20) and 21–35 years (n = 30). Protein level was measured by ELISA.

In order to investigate AMH secretion within the follicle. We obtained FF from single dominant follicle from 20 patients 40–45 years and 30 patients 21–35 years old. Looking at FF AMH protein level (Figure 2) we observed higher FF AMH levels in the older group compared to the younger one (4.7 ± 1.1 ng\ml and 2.3 ± 0.2 ng\ml respectively, p < 0.002).

### Serum AMH levels in relation to age

**Figure 3:**
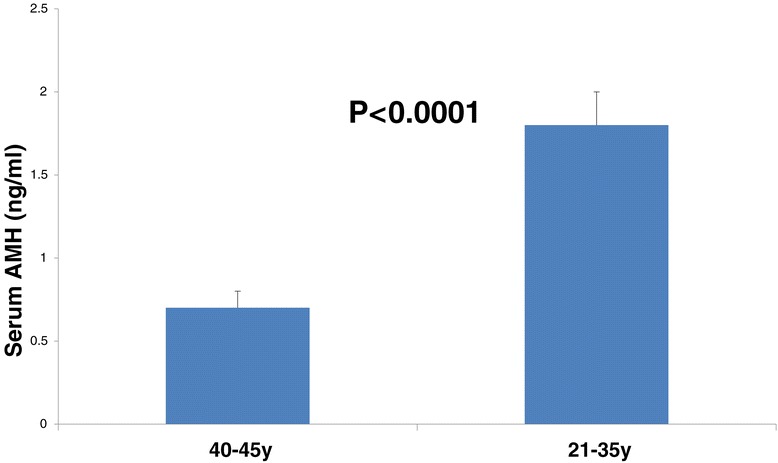
**Serum AMH level in relation to age.**

As expected, serum AMH levels were significantly higher in the younger group than in the older group (Figure 3).

## Discussion

The present study demonstrates that in elderly patients (40-45y), transcription and secretion of AMH from cumulus GCs is significantly higher than in younger patients (21-35y). However, serum AMH levels were higher in younger patient group as expected. To the best of our knowledge, this is the first study that demonstrates AMH expression levels in mural and cumulus granulosa cells and AMH protein levels in relation to age.

Serum AMH is increasingly used to asses ovarian function [14-16]. Recently, Several studies have shown that AMH expression remains high until the follicle reaches a diameter of around 8 mm [5,6,9,17]. The intrafollicular concentrations of AMH in normal human antral follicles show a gradual reduction as the diameter of the follicle increases, and a rapid decline in AMH expression corresponds with the selection of follicles for dominance. Recent data from our lab demonstrates a dysregulated AMH expression in various pathological conditions in reproduction. Such as in follicles containing immature and atretic oocytes [12] and in PCOS patients [5]. This tendency of AMH dysregulation in different pathological processes suggests that AMH may be involved in the pathogenesis of these conditions. A number of animal and human studies support this theory by demonstrating an inhibitory role of AMH in follicular fluid. In murine granulosa cells, the FSH- and cAMP-stimulated aromatase activity was significantly reduced after treatment with AMH [18]. Pellatt et al. [19] reported that AMH inhibits factors that promote follicle progression and growth in human granulosa cells. In addition, recent studies examined the correlation between FF AMH and its expression to oocyte quality, fertilization capacity and related embryo quality in large preovulatory follicles. Mehta et al. found that low follicular fluid AMH was associated with higher percentage of top-quality oocytes, fertilization, clinical pregnancy, embryo implantation rates and clinical pregnancy rates [20]. On the other hand, they are in contrast to those of Fanchin et al. who found that clinical pregnancy rates, embryo implantation rates were worse with low follicular AMH levels [21]. Thus, we hypothesized that higher FF AMH level in advanced age patients, may affect follicular health.

Not surprisingly, serum AMH levels were not affected by the increased FF AMH levels in the group of elderly patients. That may indicate that serum AMH is in direct correlation with the quantity of preantral and small antral follicles.

In summary, the present study shows a sharp increase in cumulus expression with increasing age and higher FF AMH level in the older than in the younger age groups. This remarkable correlation between AMH mRNA levels in cumulus cells in respect to age suggests that AMH may be involved in follicular aging process.
